# Maximizing the health impact of dietary fibers with microbial community-scale metabolic modeling

**DOI:** 10.1093/sumbio/qvag049

**Published:** 2026-11-11

**Authors:** Nick Quinn-Bohmann, Sean M Gibbons

**Affiliations:** Institute for Systems Biology, Seattle, WA 98109, United States; Institute for Systems Biology, Seattle, WA 98109, United States; Department of Biomedical Engineering, Tandon School of Engineering, New York University, New York, NY 11012, United States; Department of Bioengineering, University of Washington, Seattle, WA 98195, United States; Department of Genome Sciences, University of Washington, Seattle, WA 98195, United States; Department of Environmental and Occupational Health Sciences, University of Washington, Seattle, WA 98195, United States

Few interventions have the potential to improve population health as profoundly as changing what we eat, yet some of the most effective dietary patterns, many of which emphasize plant-based foods and high dietary fiber, remain stubbornly difficult to implement at scale due to a variety of economic, structural, and behavioral factors. For example, even in landmark trials such as PREDIMED, which demonstrated a reduction in cardiovascular disease risk with a Mediterranean diet, sustained adherence proved challenging. Approximately half of PREDIMED participants remained strictly adherent to their assigned diet, despite quarterly group sessions and individual in-person consultations with registered dietitians (Downer *et al*. 2016, Estruch *et al*. 2018). Although obtaining nutrients from whole foods remains preferable, supplementing prebiotic fibers into an individual’s habitual diet offers a lower-burden strategy for increasing fiber intake without requiring wholesale changes to established dietary habits (Tuohy *et al*. 2024).

Dietary fiber is, arguably, the most essential nutrient that has been depleted from the developed world diet (Reynolds *et al*. 2019). Dietary fibers are complex polysaccharides that resist digestion and absorption in the small intestine, and can confer substantial health benefits to the host (Dahl and Stewart 2015, Reynolds *et al*. 2019). These molecules are fermented by the colonic microbiota, resulting in the production of short-chain fatty acids (SCFAs), like acetate, propionate, and butyrate, which promote epithelial barrier function, proper immune function, and better physiological health (Mann *et al*. 2024). Increased consumption of dietary fiber is consistently associated with lower risk of cardiovascular disease, inflammatory bowel diseases, metabolic dysfunction, hypertension, and certain cancers (Dahl and Stewart 2015, Stephen *et al*. 2017, Reynolds *et al*. 2019). Yet despite these well-established benefits, fiber intake remains stubbornly low. Fiber intake has fallen dramatically from ancestral estimates of around 100 g per day to historically low levels of approximately 14–25 g per day in modern developed-world populations (Dahl and Stewart 2015, Stephen *et al*. 2017, Armet *et al*. 2022). In the USA, only an estimated 5% of adults meet recommended intake levels of 25 g per day for women and 38 g per day for men (Dahl and Stewart 2015). Consequently, many US adults are chronically underexposed to beneficial SCFAs.

Meta-analyses of randomized controlled trials of inulin-type fructans, a common class of prebiotic fibers, have demonstrated beneficial effects on markers of cardiometabolic health, including low-density lipoprotein (LDL) cholesterol, serum triglycerides, blood glucose, and body weight (Li *et al*. 2021, Talukdar *et al*. 2024). However, these analyses also reveal substantial unexplained heterogeneity in the magnitude of these effects, suggesting that responses to prebiotic supplementation may be highly context- and individual-dependent (Li *et al*. 2021, Talukdar *et al*. 2024). This variability is due in part to interindividual variation in gut microbiota composition, which gives rise to distinct microbial metabolic phenotypes and differences in dietary fiber utilization (Gurry *et al*. 2021). As a result, supplementing with the same dietary fiber at a population scale can elicit markedly different responses across individuals, particularly in the quantity and relative proportions of the SCFAs produced (Gurry *et al*. 2021, Kok *et al*. 2024). Consequently, a one-size-fits all approach to prebiotic supplementation may result in a substantial proportion of individuals receiving interventions to which they derive limited or no metabolic benefit (Kok *et al*. 2024).

Estimating how a microbial ecosystem with any arbitrary composition will respond to an arbitrary nutrient input has proven to be a nearly intractable problem. Machine learning approaches have been effective for small *in vitro* communities, given sufficient training data, but these models cannot be readily extended beyond the organisms and media conditions they were trained on (Clark *et al*. 2021). However, recent advances in microbial community-scale metabolic modeling (MCMM) provides a mechanistic framework well-suited to this task. MCMMs are constructed by combining curated genome-scale metabolic models (GEMs) corresponding to the taxa present in a given sample, with their relative abundances constrained by fecal metagenomic data. These individual GEMs are integrated into a single community model in which each taxon can exchange metabolites with a shared environment and with other members of the community, enabling simulation of microbial growth and metabolic interactions. The community is further constrained by an *in silico* medium, which defines the metabolites available for uptake. Growth is simulated using the cooperative tradeoff flux balance analysis approach (Diener *et al*. 2020). In brief, the community growth rate is optimized to identify the maximum achievable growth rate, after which the community is constrained to maintain a specified fraction of this maximum, defined by the tradeoff parameter. Within this constraint, the solution is selected to minimize the sum of squared individual growth rates, distributing growth across community members rather than allowing the solution to be dominated by a small number of fast-growing taxa. Results from these models take the form of metabolic exchanges (imports and exports) and predicted growth rates for each taxon in the community.

Previous work from our group has demonstrated the capacity of MCMMs to accurately predict microbial production of SCFAs in response to individual fiber supplementation, successful engraftment of probiotic species, and susceptibility of microbial communities to invasion by the opportunistic pathogen *Clostridioides difficile* (Quinn-Bohmann *et al*. 2024, 2026; Carr *et al*. 2025). Once an MCMM has been constructed for an individual, alternative prebiotic and dietary interventions can be simulated by modifying the composition of the *in silico* medium. Varying the type and quantity of dietary fiber can therefore be used to predict how different substrates alter community metabolism and metabolite production. Multiple prebiotics can likewise be evaluated in combination, allowing, for example, an inulin-type fructan to be paired with structurally distinct fibers, such as arabinoxylan or resistant starch. The optimal intervention could then be identified by evaluating model-predicted outcomes against a predefined therapeutic objective, such as maximizing predicted microbial butyrate production flux.

MCMMs can also identify individuals for whom prebiotic supplementation alone is unlikely to be sufficient to achieve a given objective, like higher levels of butyrate or propionate. For some microbiomes, model simulations may predict minimal SCFA production across a broad range of prebiotic interventions, suggesting that the limiting factor is not the availability of an appropriate substrate but the absence or insufficient abundance of microbes capable of utilizing it. In these cases, supplementing the prebiotic with a probiotic could provide both the substrate and the metabolic capacity required for its conversion into beneficial metabolites. Such combinations, termed synbiotics, could therefore be tailored to overcome specific metabolic deficiencies within an individual’s microbiota. MCMMs can be run quickly enough (a few minutes per model, run in parallel) to simulate hundreds-to-thousands of prebiotic, probiotic, and dietary combinatorial interventions for each individual in a population, allowing the model to identify relatively high-order, personalized combinations predicted to produce a desired metabolic outcome (Fig. 1a).

**Figure 1:**
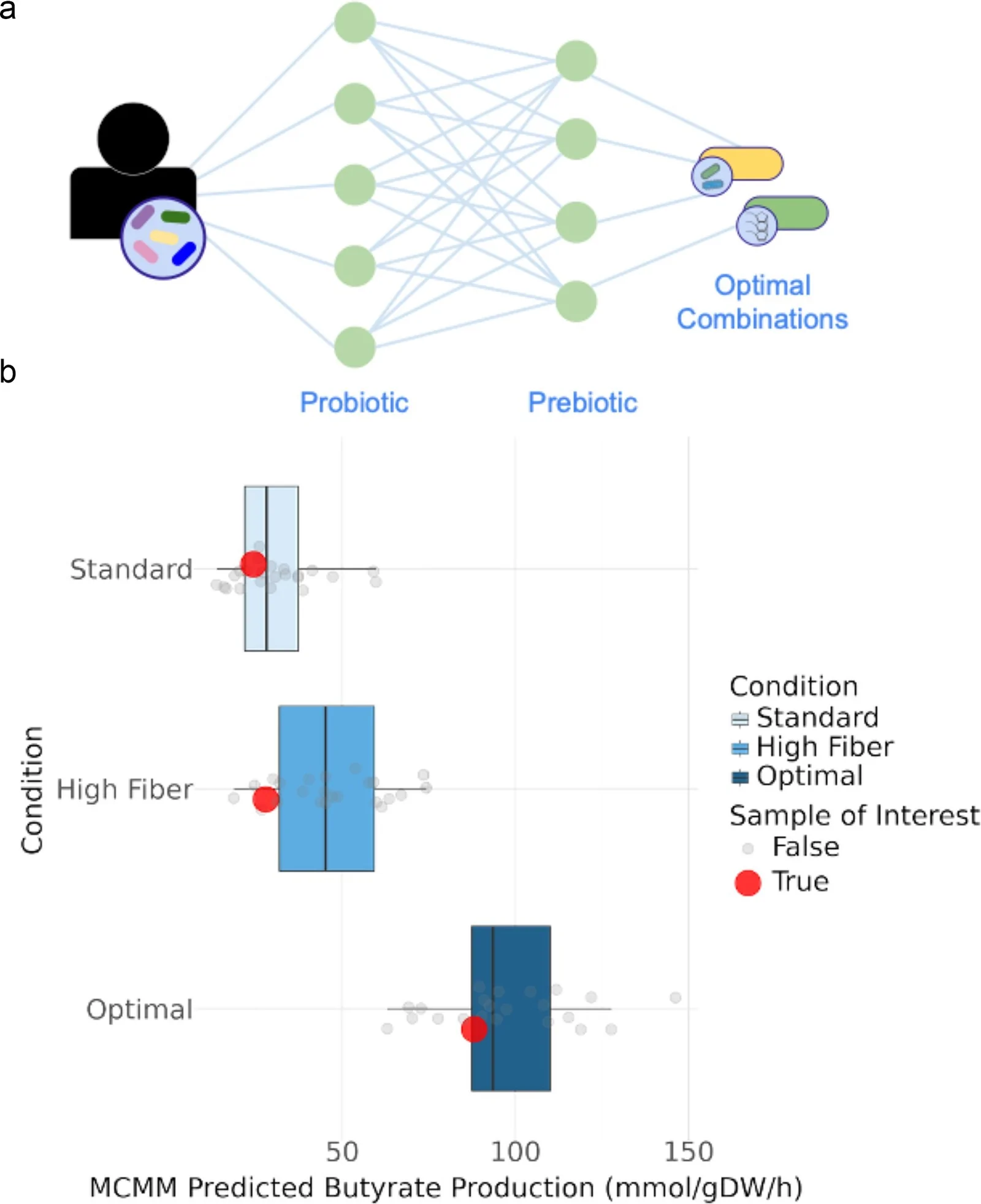
(a) MCMMs enable exploration of a large combinatorial space of prebiotic and probiotic interventions to identify combinations predicted to maximize a defined metabolic endpoint. (b) Simulation of personalized interventions selected from a combinatorial space of five probiotics and six prebiotic fibers demonstrates that personalized combinations can increase butyrate production beyond that achieved by a high-fiber diet and improve butyrate production in predicted non-responders. The larger point in each boxplot represents the same individual, who shows minimal butyrate production under both the European and high-fiber diets but a marked increase following the personalized intervention (i.e. they achieve higher butyrate production than any of the individuals on the high-fiber diet).

For example, simulations of 30 individuals predicted the expected increase in butyrate production following a switch from an individual-specific diet, based on responses to food frequency questionnaires (mean fiber intake, 16 g/day), to a high-fiber vegan diet containing 45 g fiber/day. However, several individuals showed little or no increase in butyrate production on the higher-fiber diet, which we would label as non-responders. We then tested the combinatorial space of five probiotic species and six prebiotic fibers in the context of each individual’s baseline diet, with prebiotic supplementation calibrated to provide approximately the same additional amount of fiber as the switch to the 45 g/day high-fiber diet. Across individuals, the effects of prebiotic and probiotic supplementation were generally modest when administered independently, whereas their combination produced substantially greater increases in butyrate production, highlighting the functional complementarity between substrate provision and microbial metabolic capacity. Personalized probiotic–prebiotic combinations were predicted to increase butyrate production beyond that achieved by the high-fiber diet and, importantly, to rescue the predicted butyrate response in the high-fiber diet non-responders (Fig. 1b).

Despite their promise, several limitations must be addressed before MCMMs can be implemented clinically. Because these models are constructed from genome-scale metabolic models, access to high-quality, well-validated GEMs is critical. Large and continually improving model databases are beginning to address this challenge (Heinken *et al*. 2020, 2025). Background dietary intake represents another important consideration, as differences in substrate availability can substantially alter microbial metabolism and metabolite production but are not routinely captured in current modeling approaches. Recent work has begun to address this limitation by developing methods to infer dietary intake directly from metagenomic sequencing data (Diener *et al*. 2025). Additionally, MCMMs primarily capture metabolic interactions among viable microbial community members and do not represent host-mediated effects or biological activity arising from non-viable microbial cells or their components, which may also contribute to the effects of probiotic supplementation. More broadly, model predictions that have currently undergone some degree of preclinical validation will require further experimental and prospective clinical validation before they can be translated into treatment recommendations.

The ultimate opportunity, however, is to move beyond asking which prebiotic is most effective on average across a population and instead ask which intervention is most likely to benefit a particular individual. MCMMs could identify the prebiotics, probiotics, or combinations of both, predicted to produce the most favorable metabolic response within an individual’s microbiome, while evaluating combinations that would be impossible to test exhaustively in experimental studies. In this way, MCMMs could serve as ‘‘digital twins’’ that provide a path toward microbiome-guided precision nutrition, transforming prebiotic supplementation into a personalized strategy for manipulating microbial metabolism. Finally, if we can leverage MCMMs to achieve similar metabolic benefits from personalized supplements as are currently observed in healthy dietary intervention trials (e.g. with Mediterranean-style diets), then many of the long-term adherence issues that have thwarted translation of whole food-based nutritional recommendations may be circumvented.
